# The distribution and taxonomy of *Lissotriton* newts in Turkey (Amphibia, Salamandridae)

**DOI:** 10.3897/zookeys.484.8869

**Published:** 2015-02-26

**Authors:** Ben Wielstra, Emin Bozkurt, Kurtuluş Olgun

**Affiliations:** 1 Department of Animal and Plant Sciences, University of Sheffield, S10 2TN Sheffield, UK; 2 Naturalis Biodiversity Center, P.O. Box 9517, 2300 RA Leiden, The Netherlands; 3 Adnan Menderes University, Faculty of Science and Art, Department of Biology, 09010, Efeler, Aydın, Turkey

**Keywords:** Anatolia, Bosphorus, historical biogeography, *Lissotriton kosswigi*, *Lissotriton lantzi*, *Lissotriton vulgaris schmidtleri*, Smooth newt

## Abstract

Two and perhaps three taxa of *Lissotriton* newt occur in Turkey. Their species status is controversial. The distribution of these taxa and the taxonomic status of each are reviewed and discussed. A database of 128 Turkish *Lissotriton* localities was compiled and species distribution models were constructed. We reiterate that the presence of Lissotriton (vulgaris) lantzi in Turkey is disputed and needs confirmation. The range of Lissotriton (vulgaris) kosswigi is restricted to north-western Anatolia – given the small global range of this Turkey endemic, a closer look at its conservation status is warranted. The distribution of *Lissotriton vulgaris schmidtleri* covers western Asiatic and European Turkey. The findings support an allopatric distribution of the Turkish *Lissotriton* species. We reflect on the biological significance of previously reported morphological intermediates between Lissotriton (vulgaris) kosswigi and *Lissotriton vulgaris schmidtleri* in the light of the recent proposal to recognize *kosswigi* at the species level. The available data are in line with species status for Lissotriton (vulgaris) lantzi and Lissotriton (vulgaris) kosswigi. Although *Lissotriton vulgaris schmidtleri* is a genetically diverged taxon as well, the extent of gene flow with parapatric European *Lissotriton* taxa is as yet unknown.

## Introduction

The Smooth newt *Lissotriton vulgaris* group (Amphibia: Salamandridae) is distributed in Europe and adjacent Asia (Schmidtler and Franzen 2004). The taxonomy of the group is a matter of dispute, with the inclusive taxa usually referred to as subspecies, although some of these have been occasionally regarded as specifically distinct (see Dubois and Raffaëlli 2009, Speybroeck et al. 2010). Based on the taxonomy of Babik et al. (2005) the *Lissotriton vulgaris* group consists of seven taxa, namely *ampelensis* (Fuhn 1951), *graecus* (Wolterstorff 1906), *kosswigi* (Freytag 1955), *lantzi* (Wolterstorff 1914), *meridionalis* (Boulenger 1882), *schmidtleri* (Raxworthy 1988) [following the rationale of Dubois (2007), Dubois and Raffaëlli (2009) make the case that the original name *schmidtleri* as in Raxworthy (1988) is correct, rather than the name *schmidtlerorum* introduced in Raxworthy (1990)], and the nominal species *vulgaris* (Linnaeus 1758).

**Figure 1.**
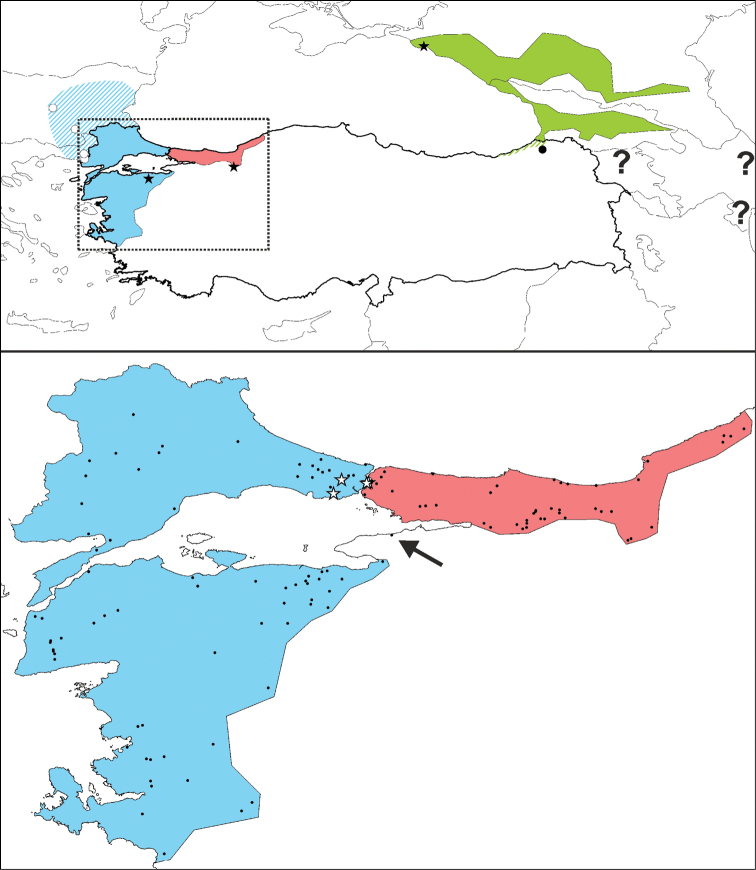
Map showing the distribution of the taxa of the *Lissotriton vulgaris* group that occur in Turkey. The inset shows the rough outlines of the ranges of *lantzi* (in green), *kosswigi* (in red) and *schmidtleri* (in blue). Type localities are marked with a black star. The blue hatched area reflects the unclear range of *schmidtleri* outside of Turkey (see discussion), with four confirmed records denoted with white dots. The green hatched area reflects the potential occurrence of *lantzi* in the extreme northeast of Turkey, with a black dot depicting the single historical record for Turkey (see discussion); question marks denote historical records in Armenia and Azerbaijan. The cut-out shows Turkish localities for *kosswigi* and *schmidtleri* as black dots. Localities supposedly showing intergradation between *kosswigi* and *schmidtleri* are marked with a white star. The arrow highlights a poorly documented locality attributed to *kosswigi* (see discussion). Details on Turkish localities are provided in Suppl. material 1.

In Turkey, two and perhaps three *Lissotriton* occur (Fig. 1; Schmidtler and Franzen 2004). The range of *lantzi* covers the Caucasus region and the taxon might occur in the extreme north-east of Turkey, near the border with Georgia (Schmidtler and Franzen 2004, Skorinov et al. 2014). The taxon *kosswigi* is restricted to north-western Anatolia (Schmidtler and Franzen 2004). The taxon *schmidtleri* was originally considered to be restricted to western Asiatic Turkey, but Raxworthy (1988, 1990) suggested it might extend into Europe. Genetic data have subsequently confirmed that this taxon’s range encompasses European Turkey (Nadachowska and Babik 2009; Pabijan et al. 2014). The range of *schmidtleri* protrudes further into the Balkan Peninsula, but its range limit is as yet unclear; newts with mitochondrial DNA typical of *schmidtleri* have been recorded as far north-west as easternmost Greece and central Bulgaria (Pabijan et al. 2014). Previous records of *vulgaris* from Turkey reflect incomplete taxonomy and can be referred to the other taxa (cf. Dubois and Raffaëlli 2012, Olgun et al. 1999).

**Figure 2.**
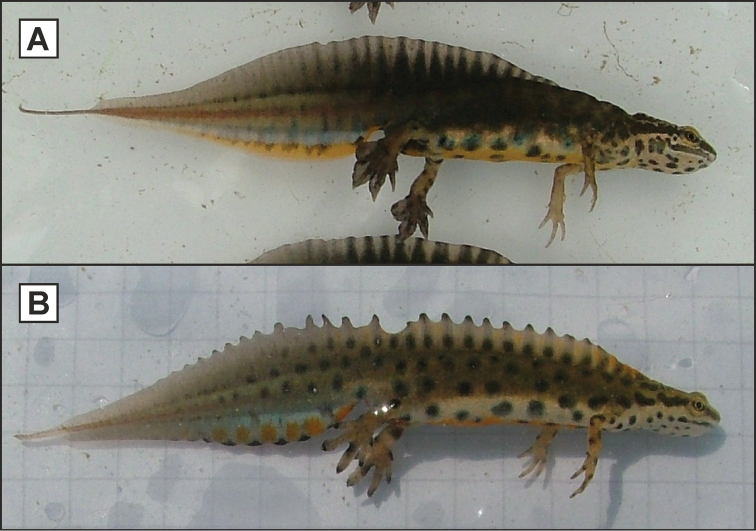
Example of the two morphological types comprising the *Lissotriton vulgaris* group of newts. Shown (not to scale) are a typical *kosswigi* male (**A**) and a typical *schmidtleri* male (**B**). Notice the shape of the doral fin (smooth in *kosswigi* and ragged in *schmidtleri*), the position where the dorsal fin starts (approximately above the forelimbs in *kosswigi* and at the back of the head in *schmidtleri*), the presence of a thread-like tail filament (found in *kosswigi* but not in *schmidtleri*) and the extensiveness of the fringing on the feet (with *kosswigi* having much more flappy feet than *schmidtleri*).

The *Lissotriton vulgaris* group comprises two main morphological types: one with a smooth crest and flappy feet and another with a ragged crest and limited fringing on the feet (Fig. 2). Distinguishing the taxa within the two main groups is less straightforward and this topic is beyond the scope of the present paper (we refer to Raxworthy (1990) and Schmidtler and Franzen (2004) for a detailed treatment). Relevant for the current paper is that *kosswigi* belongs to the 'smooth-crested with flappy feet' type and *schmidtleri* to the 'ragged-crested with limited feet-fringing' type and that morphological intergradation has been reported between these two taxa (e.g. Freytag 1955, 1957, Tabrizi 1980, Yılmaz 1983). In Fig. 2 typical males of *kosswigi* and *schmidtleri* are depicted. Next to the smooth crest and flappy feet, *kosswigi* possesses a tail filament and its crest starts at a more posterior position than in *schmidtleri*. Although *lantzi* belongs to the ‘ragged-crested with limited feet-fringing’ type as well, confusion with *schmidtleri* is ruled out based on geography.

An overview of the distribution of the Turkish *Lissotriton* taxa is provided by composing a database of localities and constructing species distribution models. The focus is mainly on the taxa *kosswigi* and *schmidtleri* and particularly the supposed genetic admixture between the two. Finally, we reflect on the as yet controversial proposal to treat the Turkish *Lissotriton* taxa as distinct species.

## Material and methods

The distribution of *Lissotriton* in Turkey has been reviewed and a database compiled of localities based on: 1) the collection of the Zoology Laboratory of the Department of Biology at Science and Arts Faculty, Adnan Menderes University, 2) extensive personal field observations, and 3) a review of the literature (Bozkurt et al. in press, Çevik et al. 1997, Çiçek and Ayaz 2011, Demirsoy 1996, Eiselt 1966, Freytag 1955, Freytag 1957, Mulder 1995, Olgun et al. 1999, Raxworthy 1988, Schmidtler and Schmidtler 1967, Skorinov et al. 2014, Sparreboom and Arntzen 1987, Tabrizi 1980, Taşkın and Olgun 2003, Yılmaz 1983, 1989). In this paper we particularly focused on *kosswigi*, this being the rarest and most restricted taxon globally. The aim was not to be exhaustive for *schmidtleri*, which is common were not included, and widely distributed in western Turkey. Localities within one kilometre of one another and in such cases the locality with the most accurate information available was chosen. We particularly focused on records of presumed transitional forms between *kosswigi* and *schmidtleri* reported in the literature, considering their relevance in the taxonomic treatment of the different *Lissotriton* taxa occurring in Turkey.

For a species distribution modelling exercise for *lantzi* (and a comprehensive overview of the distribution of this taxon outside of Turkey) we refer to Skorinov et al. (2014). Species distribution models were constructed for *kosswigi* and *schmidtleri* using Maxent 3.3.3k (Phillips et al. 2006). For climate layers bioclimatic variables were used, at 2.5 arcminute resolution (c. 5 × 5 km) available from the WorldClim database 1.4 (Hijmans et al. 2005; http://www.worldclim.org). We trimmed these layers to an extent that broadly encompasses the distribution of the genus *Lissotriton*: the area between -15 and 65 degrees longitude and between 30 and 75 degrees latitude. Following Guisan and Thuiller (2005) and Peterson (2011) a subset considered to reflect physiological limitations of the study species (in this case seasonality) was selected while showing little multicollinearity (a Pearson’s correlation of r < 0.7): bio10 = mean temperature of warmest quarter, bio11 = mean temperature of coldest quarter, bio15 = precipitation seasonality, bio16 = precipitation of wettest quarter, and bio17 = precipitation of driest quarter. To determine whether our species distribution model performs better than random expectation, we tested its AUC value against a null model based on 99 models for random localities (see Raes and ter Steege 2007 for details). Random point data were created with ENMTools 1.3 (Warren et al. 2010). To more thoroughly cover the range of environmental conditions experienced by *schmidtleri* the only four confirmed populations from outside the Turkish range (noted on Fig. 1; details in Pabijan et al. 2014) were included.

## Results

**Figure 3.**
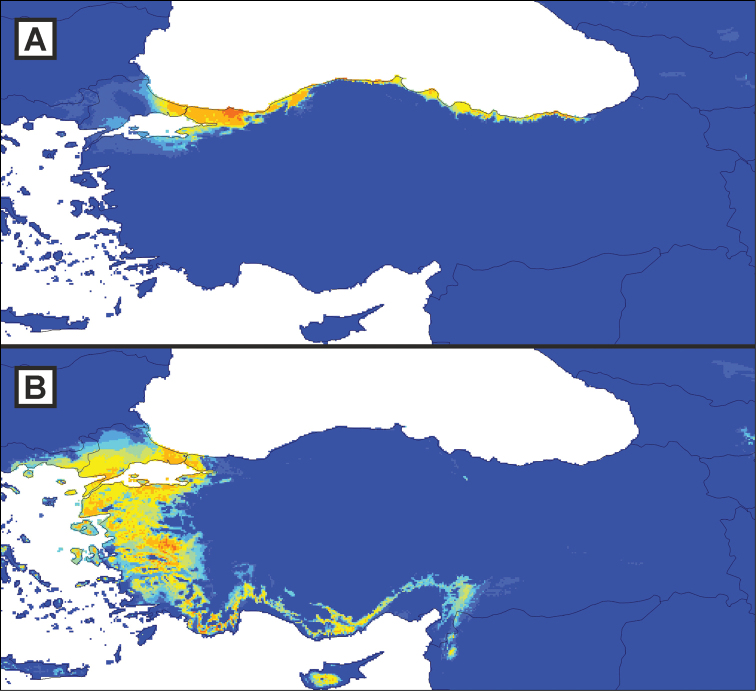
Species distribution models for two Turkish *Lissotriton* taxa. Shown are species distribution models for *kosswigi* (**A**) and *schmidtleri* (**B**). The maps depict predicted suitability, which ranges from 0 to 1, in ten equal intervals, with higher values expressed by warmer colours.

A database of 128 distribution records of Turkish *Lissotriton* newts (49 *kosswigi*, 78 *schmidtleri* and one *lantzi*) is provided in Suppl. material 1. Fig. 1 shows these records plotted on a map. The map also shows the type localities of *kosswigi* and *schmidtleri*, as well as populations reported to contain morphological intermediates between the two taxa. Fig. 3 shows the species distribution models for *kosswigi* and *schmidtleri*. The AUC values of these models (0.991 for both *kosswigi* and *schmidtleri*) rank above the 99 AUC values based on random points, meaning our species distribution models perform significantly better than random expectation (P < 0.05).

## Discussion

### Distribution

The taxon *lantzi* is widely distributed in the Caucasus region (Schmidtler and Franzen 2004, Skorinov et al. 2014). A species distribution modelling exercise (Skorinov et al. 2014) revealed that suitable environmental conditions protrude into the extreme northeast of Turkey, near the border with Georgia. However, the continued occurrence of *lantzi* in Turkey needs confirmation; there is only a single record, dating from the beginning of the twentieth century (Schmidtler and Franzen 2004, Skorinov et al. 2014). Intriguingly, there are also old reports of the Crested newt *Triturus karelinii* (Strauch 1870) from this part of Turkey (Wielstra et al. 2010). Just as *lantzi*, *Triturus karelinii* is widely distributed in the Caucasus and, although its occurrence in Turkey is suggested by species distribution modelling (Wielstra et al. 2013c), its actual presence requires further scrutiny. In any case, *lantzi* is allopatric from the other Turkish taxa: *Lissotriton* newts are absent from north-east Anatolia (Schmidtler and Franzen 2004; Fig. 1).

The distribution of the Turkey endemic *kosswigi* is restricted to north-western Anatolia (our exhaustive survey revealed 49 localities; Fig. 1). The species distribution model suggests that suitable environmental conditions extend further to the east along most of the Turkish Black Sea coast (Fig. 3). However, this area appears to be devoid of *Lissotriton* newts (Fig. 1). Over-prediction is a well-known problem in species distribution modelling (Elith et al. 2011). This could suggest that the climate layers used to create the species distribution model do not properly reflect the factors limiting the distribution of the species, but it could also suggest that not all suitable area could be colonized due to dispersal constraints.

The taxon *schmidtleri* occurs in the west of Asiatic Turkey and is now known to extend into Europe, across the marine corridor connecting the Aegean and Black Seas (Nadachowska and Babik 2009, Pabijan et al. 2014). The permeability of this apparent barrier can be ascribed to sea level fluctuations related to glacial cycles and the disjunct distribution pattern of *schmidtleri* is mirrored by the co-distributed crested newt species *Triturus ivanbureschi* Arntzen & Wielstra, 2013 in Wielstra et al. (2013a) (Wielstra and Arntzen 2012). Although the Balkan range of *schmidtleri* outside of Turkey is poorly understood, the taxon appears to occur well into Bulgaria (Pabijan et al. 2014). This is in conflict with the species distribution model (Fig. 3). It could be that mitochondrial DNA does not properly reflect the range of *schmidtleri* and overestimates its occurrence in Bulgaria. However, we consider it more likely that, due to the lack of confirmed *schmidtleri* localities from Bulgaria (whereas the taxon might well be abundant there), the species distribution model underestimates the environmental space inhabited by *schmidtleri*.

The taxa *kosswigi* and *schmidtleri* currently appear allopatric. We have particularly surveyed the area for *Lissotriton* (pers. obs.) and no localities are known between the *schmidtleri* locality Gemlik (Olgun et al. 1999; locality 62 in Suppl. material 1) and *kosswigi* locality Yalova (Demirsoy 1996; locality 18 in Suppl. material 1). The Yalova locality lacks documentation and needs confirmation (note that the locality was not included in Schmidtler and Franzen 2004) and it is suggested that there is probably a larger distribution gap, with the next closest *kosswigi* locality from the perspective of *schmidtleri* being Kocaeli (museum record; locality 3 in Suppl. material 1). This apparent distribution gap disagrees with the species distribution models, which suggest suitable environmental conditions for both *kosswigi* and *schmidtleri* occur south of the Marmara Sea (Fig. 3).

Based on introgression of *schmidtleri* mitochondrial DNA into *kosswigi* (very similar to mitochondrial DNA found in *schmidtleri* today) it has been hypothesized that *kosswigi* displaced *schmidtleri* on the Istanbul Peninsula as the waterway between the Black and Marmara Seas rerouted within the last 10,000 years (Nadachowska and Babik 2009, Wielstra et al. 2013b). Similarly, an as yet undescribed *Triturus* species was proposed to have displaced *Triturus ivanbureschi* in this region (Wielstra et al. 2013a, 2013b). The species distribution models suggest suitable environmental conditions here for both *kosswigi* and *schmidtleri* and hence do not provide further insight into how *kosswigi* was able to locally outcompete *schmidtleri* (Fig. 3).

### Genetic admixture

In light of the current allopatric distribution pattern of *kosswigi* and *schmidtleri*, previous reports of transitional forms are curious. Following up on a possible intermediate specimen from Sapanca, Eiselt (1966) could only confirm the presence of pure *kosswigi* there. Freytag (1955) indicated that in a *Lissotriton* population from Kanlıca (locality 37 in Suppl. material 1), on the eastern side of the Bosphorus, some males showed characteristics of *schmidtleri*, namely the dorsal fin being ragged and starting at the back of the head and the lack of a tail filament (cf. Fig. 2). Tabrizi (1980) studied a larger sample of newts from populations throughout the range of *kosswigi*. He found that four out of 70 newts in Kanlıca showed a *schmidtleri*-like, relatively anterior starting position of the dorsal fins; all other newts were classified as typical *kosswigi*. Considering the biogeographical scenario outlined above, a relict *schmidtleri* population in the process of being replaced by *kosswigi* via genetic swamping is a possibility. A study on historical gene flow between the two taxa unfortunately did not include samples from the potentially admixed populations, but did suggest ancient gene flow from *schmidtleri* into *kosswigi* (Nadachowska and Babik 2009).

Furthermore, Freytag (1957) mentioned that in a *Lissotriton* population from Baltalimanı (locality 104 in Suppl. material 1), on the western side of the Bosphorus, some males shared similarities with *kosswigi*, in terms of possessing tail filaments and smooth dorsal fins that started relatively posteriorly (Fig. 2). Yılmaz (1983) studied a larger sample encompassing more populations from European Turkey. He noted newts with *kosswigi* characteristics at Habibler and Küçükçekmece (localities 121 and 125 in Suppl. material 1). Out of 80 studied newts, 20 had dorsal fins that began at the forelimbs rather than at the back of the head, 41 had smooth dorsal fins and 37 had tail filaments to varying degree (17 with 0–2 mm, 10 with 2–4 mm, 5 with 4–6 mm, and 5 with over 6 mm). Schmidtler and Franzen (2004) state that in *schmidtleri* males can show *kosswigi*-like characteristics, but do not provide further details. The presence of *kosswigi* west of the Bosphorus would not make sense in light of the biogeographical scenario outlined above, unless it could be proven that the Bosphorus on initial formation had a more westward position or formed only after *kosswigi* reached European Turkey. The rerouting of the marine connection between the Marmara and Black Seas is not yet fully understood and a matter of debate in the paleogeological literature (e.g. Nazik et al. 2011, 2012, Yaltırak et al. 2012). We suggest that historical biogeographical patterns such as shown by *Lissotriton* (and *Triturus*) newts might assist paleogeological reconstruction.

Genetic data from the potentially admixed *kosswigi* and *schmidtleri* populations are as yet lacking, but would provide more insight in the matter. However, considering the expansion of the Istanbul agglomeration it should be taken into account that these populations might well have gone extinct. We conclude that potential *kosswigi*-*schmidtleri* admixture represents, at most, the remnants of a former contact zone. The main ranges of the two taxa are currently isolated in the region by the Bosphorus and hence the influence of potentially admixed populations on the genetic integrity of the two taxa can be expected to be negligible. In this light we make some remarks on the not (yet) generally accepted treatment of the Turkish *Lissotriton* taxa as distinct species (Dubois and Raffaëlli 2009, Frost 2014).

### Taxonomy

Following the taxonomy of Babik et al. (2005), the *Lissotriton vulgaris* group consists of seven taxa, namely *ampelensis*, *graecus*, *kosswigi*, *lantzi*, *meridionalis*, *schmidtleri* and the nominal *vulgaris*. Four of these taxa, *graecus*, *kosswigi*, *lantzi* and *meridionalis*, are sometimes regarded as specifically distinct (Dubois and Raffaëlli 2009, Frost 2014). The split of *graecus* and *meridionalis* has been criticised (Speybroeck et al. 2010) as a misinterpretation of the phylogenetic position of the congener *Lissotriton montandoni* which, due to mitochondrial DNA introgression, is nested within the *Lissotriton vulgaris* group from the perspective of mitochondrial DNA (Babik et al. 2005, Zieliński et al. 2013). However, the taxa *kosswigi* and *lantzi* are genuinely genetically diverged for mitochondrial DNA (Babik et al. 2005).

Within the *Lissotriton vulgaris* group mitochondrial DNA suggests a basal split between *lantzi* and the rest (Babik et al. 2005). Although the distinction of *lantzi* from the perspective of the nuclear genome has as yet not been determined, the divergence in the mitochondrial genome and the at least currently disjunct distribution support a scenario of long-term disrupted gene flow with other *Lissotriton* newts.

The next split in the *Lissotriton vulgaris* group is between *kosswigi* and the remaining taxa (Babik et al. 2005). The distinction of *kosswigi* from its geographical neighbour *schmidtleri* has been supported in a study exploring gene flow based on eight nuclear DNA markers (Nadachowska and Babik 2009). Given that *kosswigi* is genetically distinct and currently allopatric from other *Lissotriton* taxa, its treatment at the species level seems justified. From the conservation perspective it is important whether this geographically restricted, Turkish endemic is treated as a ‘unique species’ or ‘merely a subspecies’.

Although *schmidtleri* represents a distinct mitochondrial DNA clade as well, it is genetically nested within the European *Lissotriton* taxa (Babik et al. 2005, Pabijan et al. in prep.). The phylogeography of *Lissotriton* on the Balkan Peninsula is highly complex, with morphologically distinct subspecies being highly polyphyletic from the mitochondrial DNA perspective (Babik et al. 2005, Pabijan et al. in prep.). Furthermore, no doubt in part because of its turbulent taxonomical history, the morphological distinctiveness of *schmidtleri* is not well understood (Schmidtler and Franzen 2004). Hence, we refrain from making further comments on the taxonomic status of *schmidtleri* and rather await further research on nuclear gene flow between *schmidtleri* and the other *Lissotriton* taxa on the Balkan Peninsula.

## Supplementary materials

Supplementary material 1 Locality data for Turkish *Lissotriton* newts Data type: ZIP archive Explanation note: The ZIP archive contains the locality database as an Excel file and a shape file. This dataset is made available under the Open Database License (http://opendatacommons.org/licenses/odbl/1.0/). The Open Database License (ODbL) is a license agreement intended to allow users to freely share, modify, and use this Dataset while maintaining this same freedom for others, provided that the original source and author(s) are credited.Ben Wielstra, Emin Bozkurt, Kurtuluş Olgun
